# A Novel Multi-Feature Fusion Method for Classification of Gastrointestinal Diseases Using Endoscopy Images

**DOI:** 10.3390/diagnostics12102316

**Published:** 2022-09-26

**Authors:** Karthik Ramamurthy, Timothy Thomas George, Yash Shah, Parasa Sasidhar

**Affiliations:** 1 Centre for Cyber Physical Systems, School of Electronics Engineering, Vellore Institute of Technology, Chennai 600127, India; 2 School of Computer Science and Engineering, Vellore Institute of Technology, Chennai 600127, India; 3 School of Electronics Engineering, Vellore Institute of Technology, Chennai 600127, India

**Keywords:** gastrointestinal diseases, CNN, squeeze and excitation, HyperKvasir dataset

## Abstract

The first step in the diagnosis of gastric abnormalities is the detection of various abnormalities in the human gastrointestinal tract. Manual examination of endoscopy images relies on a medical practitioner’s expertise to identify inflammatory regions on the inner surface of the gastrointestinal tract. The length of the alimentary canal and the large volume of images obtained from endoscopic procedures make traditional detection methods time consuming and laborious. Recently, deep learning architectures have achieved better results in the classification of endoscopy images. However, visual similarities between different portions of the gastrointestinal tract pose a challenge for effective disease detection. This work proposes a novel system for the classification of endoscopy images by focusing on feature mining through convolutional neural networks (CNN). The model presented is built by combining a state-of-the-art architecture (i.e., EfficientNet B0) with a custom-built CNN architecture named Effimix. The proposed Effimix model employs a combination of squeeze and excitation layers and self-normalising activation layers for precise classification of gastrointestinal diseases. Experimental observations on the HyperKvasir dataset confirm the effectiveness of the proposed architecture for the classification of endoscopy images. The proposed model yields an accuracy of 97.99%, with an F1 score, precision, and recall of 97%, 97%, and 98%, respectively, which is significantly higher compared to the existing works.

## 1. Introduction

The gastrointestinal (GI) tract is a tubular passage through which ingested food travels from the mouth to the anus. In anatomy, the GI tract is divided into two major portions. The ‘upper’ GI tract runs from the mouth to the duodenum, while the ‘lower’ GI tract consists of the portion from the small intestine to the anus. The upper GI tract is chiefly responsible for the swallowing and breaking down of food. Gastric acids and enzymes present in this region are responsible for the digestion of food before it is passed on to the lower GI tract. The lower GI tract furthers the digestive process with the small intestine. Water, nutrients, and electrolytes are absorbed in the large intestine. The remaining material forms a solid waste that is stored in the rectum, which later leaves the body through the anus. 

The GI tract is also susceptible to various medical conditions, which may need to be examined by medical professionals. These include gastrointestinal diseases, tissue inflammations, and abnormal growth. For example, acid reflux can cause alterations in the oesophagus lining, an abnormal immune response may trigger inflammations, or dividing cells may clump to form a polyp on the colon lining. These abnormalities can be serious themselves, as in the case of ulcers or sores. Another possibility is the development of complications at a later stage, as with polyps that may become malignant. Medical professionals demand visual examination of organs present in the GI tract.

Examination of the GI tract usually takes place with a procedure known as endoscopy [1]. These techniques involve an instrument known as an endoscope, a long and flexible tube usually attached to a fibre-optic camera, inserted through an opening. This provides a medical practitioner visual access to the GI tract, and they must then identify abnormalities while the endoscope travels the GI tract. Colonoscopy is an endoscopic technique where an endoscope is inserted through the anus for an examination of the colon or large intestine. Other types of endoscopic techniques include wireless capsule endoscopy (WCE) and narrow band imaging (NBI).

Medical image classification is an important area of image recognition and aims to help medical professionals provide diagnoses on the disease. Computer vision and artificial intelligence can assist medical professionals in the process of classifying endoscopic images into different classes. Computational endoscopic image analysis consists of four stages: pathology detection, pathology classification, pathology localisation, and pathology segmentation [2]. Researchers have been working on different learning models to carry out accurate classification of gastrointestinal images. This work presents an automatic deep-learning-based approach for classification of different GI diseases.

## 2. Related Work

Deep learning and machine learning methods have been extensively applied for the classification of biomedical images. These classification approaches aid the medical professionals to perform accurate diagnosis and devise precise treatment plans. This section reviews the existing methods applied to the classification of GI diseases.

### 2.1. Machine Learning Methods

In this section, we have discussed different machine-learning-based methods for endoscopic image classification. These methods include classification algorithms like naive Bayes, decision tree (DT), random forest, support vector machine (SVM), and so on.

Sivakumar et al. proposed an approach for the automatic classification of obscure bleeding using superpixel segmentation and naive Bayes classifier [3]. The features extracted include area, centroid, and eccentricity of the segmented region. The expectation-maximisation method was applied as the learning method in this work. Hassan et al. presented an approach to detect gastrointestinal haemorrhage from WCE images using normalised grey level co-occurrence matrix (NGLCM) features [4]. These features were trained using SVM for classification. Ali et al. utilised hybrid texture features based on Gabor transform for the detection of gastric abnormalities [5]. Gabor-based grey-level co-occurrence matrix-based features were extracted from the input chromoendoscopy images and classified using SVM.

Jani et al. presented an ensemble approach for the classification of capsule endoscopy images [6]. Colour, texture, and wavelet moments were extracted as features and trained with the ensemble classifier involving k-nearest neighbour (KNN) and SVM. Charfi et al. proposed another approach based on texture features for the detection of colon abnormalities from WCE images [7]. Discrete wavelet transform was applied to the input image and local binary pattern-based features were extracted. SVM and multilayer perceptron was used for classification. 

In addition to the methods discussed above, few research works have employed invariant features for classification of GI diseases. Moccia et al. presented an approach for the classification of laryngoscopic images [8]. Eight different types of features were employed as descriptors for each frame and classified using SVM. Another approach based on invariant features for the classification of Barrett’s oesophagus and adenocarcinoma was presented by Luis et al. [9]. Invariant features were extracted using three algorithms, namely scale-invariant feature transform, speeded-up robust features, and accelerated KAZE. These features were finally classified using the optimum path forest classifier. All the above methods analyse different types of features to detect and classify GI diseases. Al-Rajab et al. presented an approach for the classification of colon cancer using SVM [10]. Optimisation was carried out using genetic algorithms and particle swarm optimisation to yield improved performance.

The performance of the machine learning methods discussed above relies heavily on the precise identification and selection of distinct features from the input endoscopy images. This requires significant domain expertise involving gastroenterology. 

### 2.2. Deep Learning Methods

Deep-learning-based approaches are applied for different machine-based tasks in the field of healthcare for the classification of images. The CNN models are fully connected neural networks that develop a certain perception of the class of disease through a layered stack of learnable units. It can comprehensively analyse the features of different GI diseases in endoscopic images for precise classification. CNN has been employed to perform GI image classification in many research works [11,12,13]. These works utilise different types of architectures such as AlexNet [11], LSTM [14], and U-Net [13]. Igarashi et al. employed the AlexNet architecture to classify fourteen categories of upper gastrointestinal regions associated with gastric cancer [11]. Another work proposed, for the classification of wireless capsule endoscopic images, using two different models, namely ResNet and DenseNet [15]. It was reported that the DenseNet network yielded optimal results after fine tuning the model. KahsayGebreslassie analysed the performance of ResNet50 and DenseNet121 models to classify eight GI diseases [12]. It was reported that Res Net50 model was able to outperform DenseNet121 model. Rather than training separate networks, Rahman et al. used a combination of three architectures, namely Xception, ResNet-101, and VGG-19 [16]. It was reported that the ensemble architecture was effective in classifying the input images when compared to the individual networks. 

Another feature fusion model was proposed by Zeng et al. for the classification of ulcerative proctitis [17]. Ensembling was performed with three different networks, namely Xception, ResNet, and DenseNet. Ellahyani et al. proposed an ensemble approach for polyp detection from colonoscopy images [18]. Lafraxo et al. proposed a transfer-learning-based approach for the classification of endoscopic images [19]. It involved the application of MobileNet, VGGNets, and InceptionV3 architecture. He et al. proposed an approach that used pretrained ResNet-152 and MobileNetV3 to classify gastrointestinal diseases using the HyperKvasir dataset [20]. This work involved a two-stage approach for detection and segmentation. Another approach was proposed using the application of MobileNetV2 and ResNeXt-50 models for an imbalanced dataset [21]. Barbhuiya et al. employed a tiny darknet model for the detection of lesions from endoscopic images [22]. All the above-discussed methods employ pretrained networks for feature extraction. The performance of these methods can be further improved by applying appropriate customisations to these models for precise feature learning. 

Ozturk et al. presented a residual long short-term memory (LSTM) model for the classification of GI diseases [14]. It was reported that the residual LSTM structure outperformed the state-of-the-art methods in terms of classification performance. Zhao et al. proposed an abnormal feature attention network for the classification of GI endoscopy images [23]. This network leveraged the significance of few-short learning to obtain improved performance. Luo et al. proposed another attention-based deep learning network for the diagnosis of ulcerative colitis [24]. This network utilised the spatial and channel attention mechanisms on top of DenseNet to obtain better results. In addition to the above-discussed networks, custom CNNs were also proposed for the classification of GI diseases. 

Wang et al. proposed a three-stream classification network for esophageal cancer [25]. This network involved hybrid hessian filtering for preprocessing the images. Iakovidis et al. proposed a weakly supervised deep learning network for the detection and localisation of GI anomalies [26]. It employed the concepts of deep saliency detection and iterative cluster unification for precise detection and localisation. Cao et al. proposed an attention-guided network for the classification of WCE images [27]. Global and local features were extracted from the input images and refined using the attention feature fusion module. Rahim et al. proposed a deep CNN for the detection of polyps from colonoscopy images [28]. This network consisted of sixteen convolutional layers with Mish and ReLU activations. Hatami et al. presented a deep learning network for the detection and classification of gastric and precancer diseases [29]. This network involves squeeze and excitation mechanisms for improved performance. Galdran et al. proposed a hierarchical approach for the analysis of GI images. This network utilised double autoencoders for the segmentation of polyps. Gjestang et al. proposed a teacher-student framework for the classification of GI diseases [30]. This network utilises unlabelled data for better generalisation.

Jin et al. proposed a convolutional multilayer perceptron encoder for accurate polyp segmentation by considering the low-level features using a parallel self-attention module [31]. Ji et al. presented video-based polyp segmentation using a network called SUN-SEG [32]. The network was designed with novel elements such as global and local encoder and normalised self-attention blocks. Zhang et al. presented an adaptive context selection method for precise segmentation of polyp structures [33]. All the above methods present the efficacy of the deep learning models for the detection and segmentation of GI diseases. 

### 2.3. Research Gaps and Motivation 

The following are the research gaps observed with respect to the classification of GI diseases:

1. Most of the research works for the classification of GI diseases were conducted with limited datasets due to privacy concerns and the rarity of abnormalities. Hence, to improve the effectiveness of these models, these methods must be analysed with benchmark datasets.
2. Though the cumulative performance of the above-discussed methods were considerable, the class-wise metrics are often overlooked. This is due to the similarity in the morphological features existing between two or more diseases. Identification of precise hand-crafted features is a challenging task. Hence, the power of deep learning methods needs to exploited in the place of machine learning methods, which require manual feature extraction.
3. Though few deep learning research works were already reported for GI disease classification, these methods were restricted to pre-trained models or a fusion of pre-trained models. Hence, there exists a vital need to apply suitable architectural enhancements/customisations to these models for improved performance.

The following are the research contributions made toward addressing these gaps:

1. The proposed experiments were conducted with the HyperKvasir benchmark dataset for better generalisation of all classes. This ensures that the proposed method is evaluated for a benchmark dataset with 23 different classes of GI.
2. The proposed research presents a hybrid deep learning approach involving a pre-trained network and a custom CNN. While EfficientNet B0 was applied on one track to extract prominent features, custom CNN was employed on the other side for effective feature calibration. Finally, the features from both networks are fused to represent a high context feature vector representing each class.
3. We have proposed an effective feature extractor namely ‘Effimix’. It involved the application of squeeze and excitation layers, background elimination, and a non-monotonic activation function. By combining the features from Effimix and EfficientNet B0 backbone, the proposed feature fusion network was able to achieve good inter-class metrics.

## 3. Proposed System

**Figure 1:**
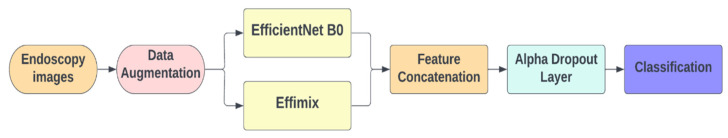
Architectural overview of the proposed system.

Figure 1 presents an architectural overview of the proposed system. A wide interest has been observed in medical research that interprets gastrointestinal images using artificial intelligence (AI) algorithms. This research proposes an automated classification technique based on deep learning to classify different gastrointestinal diseases. The HyperKvasir labelled images dataset is used to train the proposed models. The input images are initially augmented to increase the number of samples for better generalisation. These augmented samples were fed to two independent networks, namely EfficientNet B0 and the proposed Effimix network. The features from these two networks were combined using feature concatenation and subjected to dropout regularisation. The proposed work classifies the input gastrointestinal images into 23 classes.

### 3.1. Dataset Description

**Figure 2:**
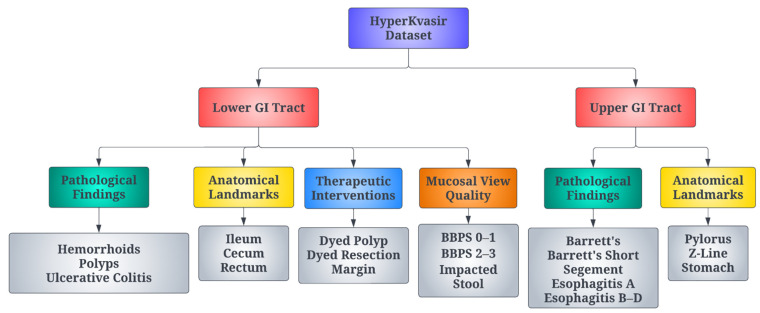
Structure of the dataset used for classification.

The proposed research has utilised 10,662 labelled images from the HyperKvasir dataset [34]. This is an open access dataset and licensed under a Creative Commons Attribution 4.0 International (CC BY 4.0). This dataset has 23 classes of data representing different gastrointestinal diseases. The input images were provided in JPEG format. The distribution of samples under each class is presented in Table 1. It could be observed that the distribution of images under different classes is highly imbalanced. The structure of the dataset has been described in Figure 2.

### 3.2. Data Augmentation 

**Figure 3:**
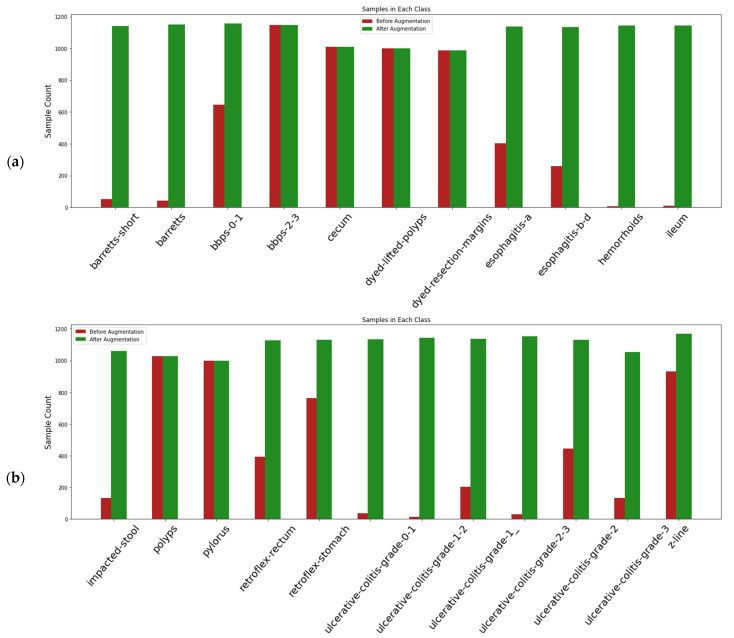
(**a**,**b**) Distribution of samples in each class before and after data augmentation.

To perform model fitting on a large dataset, the endoscopic images in the dataset were augmented by applying four random geometric transformations: horizontal flip, width shift, height shift, rotation. The parameters for the transformation function for rotation is 45°. ∆x and ∆y are the shifts for width and height, set within 0.3 of the original images. The fill-mode parameter was used for horizontal flip. By doing so, the number of input images also increases considerably. Data augmentation was performed to handle the class imbalance associated with the dataset. After augmentation, a total of 23,000 images were generated through data augmentation, and the class-wise distribution of the samples are highlighted in Figure 3. 

### 3.3. EfficientNet B0

Tan et al. proposed a novel family of models known as the EfficientNets in the year 2019 [35]. Refinements to network width, depth, and image resolution were performed to achieve higher accuracy than existing ConvNet models. The baseline network reported in the previous study was EfficientNet B0. It contains 5.3M parameters, the fewest in the EfficientNet family. EfficientNet B0 network relies on squeeze and excitation layers and an inverted bottleneck block called MBConv. The MBConv block was originally introduced in the MobileNetV2 model to improve parameter reduction [36].

### 3.4. Effimix

**Figure 4:**
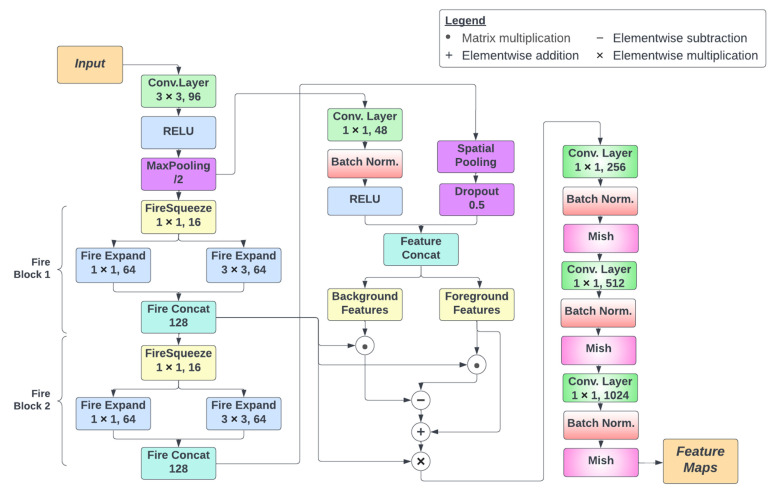
Architectural overview of the Effimix network.

The overall architecture of the proposed Effimix network is presented in Figure 4. It consists of three different processing stages. The first stage involves the application of squeeze and excitation layers. These layers are used to improve the representational power of the network by enabling it to perform dynamic channel-wise feature recalibration. In this network, two fire blocks are involved to implement squeeze and excitation. Each fire block consists of a squeeze convolution layer (which has only 1 × 1 filters), feeding into an expand layer that has a mix of 1 × 1 and 3 × 3 convolution filters. The output of the expand layer is fed into a concatenation layer that combines the feature maps derived from the previous layers. 

The second stage involves the application of background erasing and foreground mining. The concept of background erasing and foreground mining is inspired from DSI-Net [37]. This stage uses the features from the first fire block as a base feature map. Foreground features are extracted from the foreground mask, from which high-confidence feature vectors are selected to represent the foreground areas. Similarly, mined background areas are represented by high confidence feature vectors extracted. Both the background and foreground vectors are passed through a binary gated united (BGU) to reduce noise. Then, to the base feature map, the background features are subtracted, and foreground features are added. The resulting feature map provides a better input to the classification layer. 

The final stage involves a series of convolutional operations for classification. These layers include convolution and batch normalisation, followed by a non-monotonic activation function, Mish [38]. 

The Mish activation function was employed to achieve a deeper propagation of information across the network and to avoid saturation during training. The relation for Mish activation is presented in Equation (1).

(1) fx=x.tanh softplusx , where softplusx=ln1+e^x

The final output of the Effimix model will be a set of distinct feature maps, which enable precise classification of gastrointestinal diseases.

### 3.5. Classification

The feature maps extracted from the two models, namely EfficientNet B0 and Effimix, are combined using feature concatenation. These features were subject to alpha dropout regularisation for final classification. The alpha dropout layer is responsible for randomly setting activations to a negative saturation value. This ensures the self-normalising property of the model. The mean and variance after the alpha dropout are given in Equations (2) and (3), respectively.

(2) $$E\;\left(xd+\alpha^{\prime }\left(1-d\right)\right)=qµ+\left(1-q\right)\alpha^{\prime }$$

(3) $$Var\;\left(xd+\alpha^{\prime }\left(1-d\right)\right)=q(\left(1-q\right)\left(\alpha^{\prime }-µ{)}^{2}+v\right)\;$$

where x is an activation, q is a probability value in the range  0<q ≤ 1, μ is the mean before dropout, v  is the variance before dropout, d is the dropout variable, and $\;\alpha^{\prime }$ is the random values set by the dropout function.

Following the alpha dropout layer, these feature vectors are subjected to dense layers followed by softmax activation to classify 23 gastrointestinal diseases.

## 4. Results and Discussion

This section discusses the environmental setup that was used to train the proposed models. It also provides an overview of the different ablation studies carried out as part of this research. Finally, it compares the performance of the proposed model against the existing works.

### 4.1. Environmental Setup

The proposed network was trained on two 12 GB NVIDIA Tesla K80 GPUs available on Google Cloud VM. The implementation of the network was done with the PyTorch framework. The model was trained with the Adam optimiser, with a learning rate of 0.0001 and a weight decay of 0.0001. To train the data, 15,264 images in the training data set were divided into 954 batches, each with 16 images. Certain classes in the original data set had very few images available for training. Hence, data augmentation was employed to address the class imbalance existing in the input dataset. In addition, we have adopted a sampling approach to admit an equal number of images from each class for all batches during training. 

### 4.2. Ablation Studies

In this section, we will discuss the importance and effectiveness of different components employed as part of the proposed network. The network benefits from the contributions made by the different modules, which are explained in the forthcoming subsections.

#### 4.2.1. Analysis of the EfficientNet B0 Network

**Figure 5:**
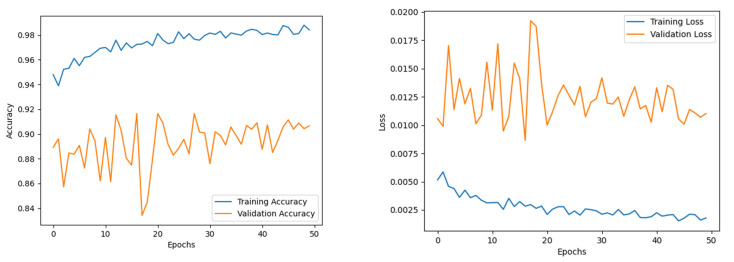
Accuracy and loss of EfficientNet B0.

The EfficientNet B0 model was trained for 50 epochs to ensure convergence of the different sub-modules. The Adam optimiser was used, with a learning rate and weight decay of 0.001 and 0.0001, respectively. The training accuracy experienced a steady increase throughout the training. While the validation accuracy trajectory staggered mid-training, its value fluctuated around a ±6 interval around the 90% band. The model presented an accuracy of 95.6%, with an F1 score, precision, and recall of 95%, 95%, and 95%, respectively. The epoch-wise accuracy and loss of the EfficientNet B0 model is presented in Figure 5.

#### 4.2.2. Analysis of the Effimix Network

**Figure 6:**
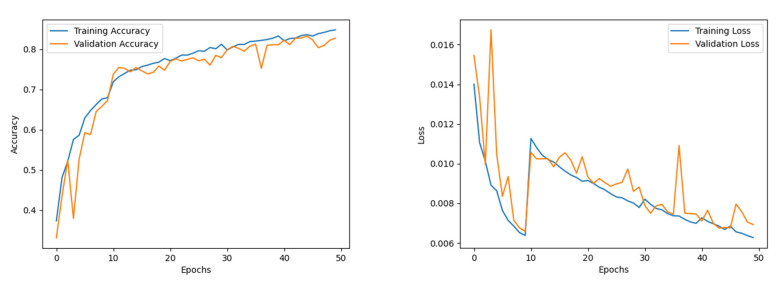
Accuracy and loss of Effimix model.

The Effimix model was trained for 50 epochs to ensure convergence of the different sub-modules. The Adam optimiser was used, with a learning rate and weight decay of 0.0001 and 0.0001, respectively. The training accuracy experienced a steady increase throughout the training. While the validation accuracy staggered in the initial part of the training, it was progressively increasing until it showed signs of saturation around the 40th epoch. The model presented an accuracy of 85.4%, with an F1 score, precision and recall of 85%, 85%, and 85%, respectively. The epoch-wise accuracy and loss of the Effimix model is presented in Figure 6.

#### 4.2.3. Analysis of the Proposed Feature Fusion Network

**Figure 7:**
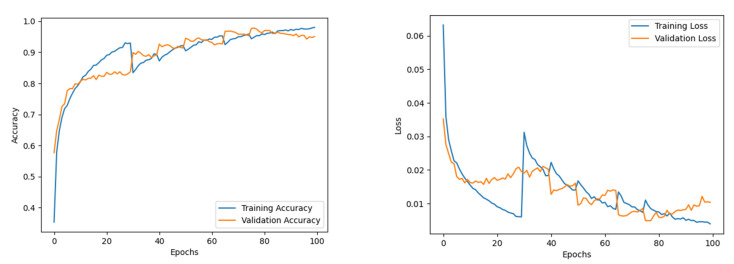
Accuracy and loss of combined model.

**Figure 8:**
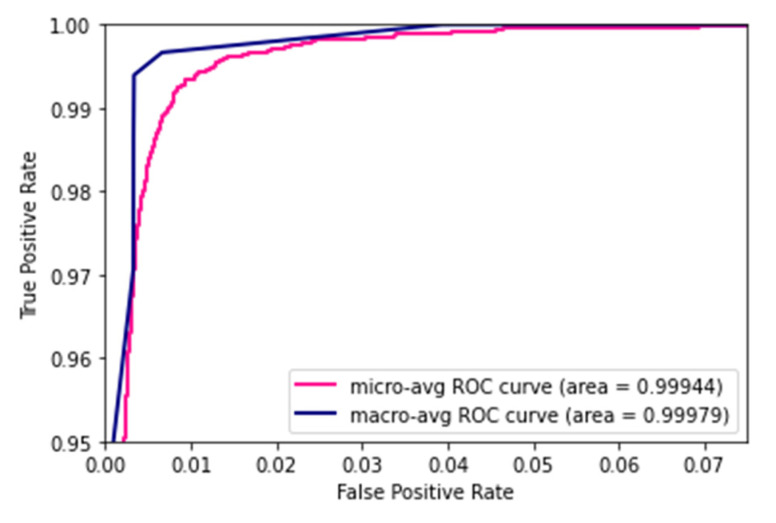
ROC plot.

**Figure 9:**
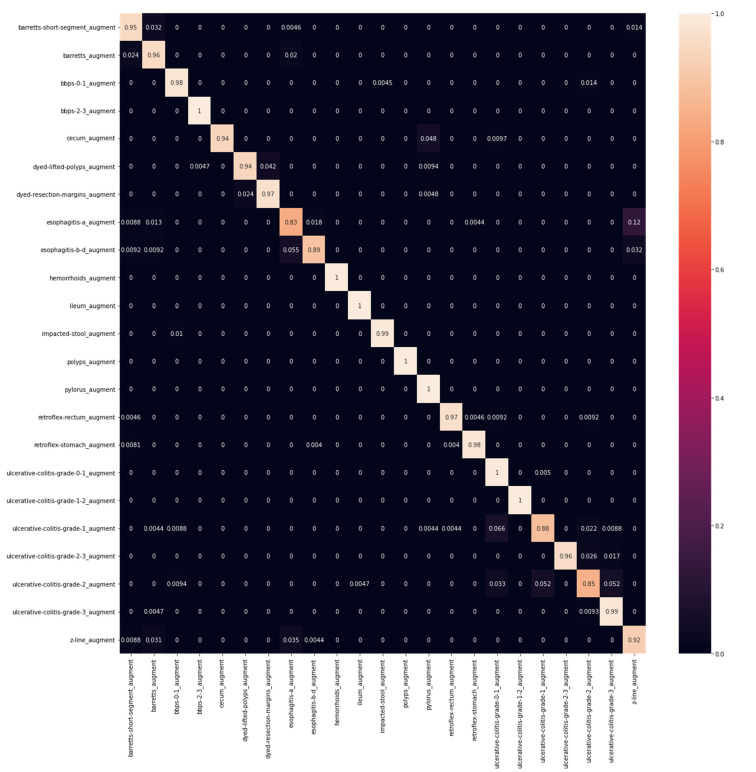
Confusion matrix.

The feature maps from the EfficientNet B0 were combined with the Effimix network to improve the feature representation power of the proposed network. This combined model was trained for 100 epochs to ensure convergence of the different sub-modules. The Adam optimiser was used, with a learning rate and weight decay of 0.0001 and 0.0001, respectively. The training accuracy showed signs of saturation around the 60th epoch and stabilised around the 80th epoch during the training. While validation accuracy experienced staggered changes mid-training, it progressively increased throughout the training. The model presented an accuracy of 97.99%, with an F1 score, precision, and recall of 97%, 97%, and 98%, respectively. The epoch-wise accuracy and loss is presented in Figure 7. The receiver operator characteristic (ROC) plot obtained for the proposed systems is presented in Figure 8, and the area under curve (AUC) obtained is 0.977. In addition, the Mathew’s correlation coefficient (MCC) and the kappa scores obtained for the proposed networks are 0.9806 and 0.9807, respectively. The confusion matrix obtained for the test set is presented in Figure 9. 

A summary of the ablation studies made is presented in Table 2. Table 3 depicts the class-wise metrics of the proposed model. As can be observed from Table 3, classes such as ‘Esophagitis-a’, ‘Ulcerative Colitis Grade 2′, and ‘Cecum’ achieved low F1 scores when the EfficientNet B0 model was trained on them. A similar observation can be made for the Effimix model with the ‘Baretts-short’, ‘Esophagitis-a’, and ‘Cecum’ classes. However, the F1 scores of these classes have been increased significantly when the combined model was trained on them. Thus, our combined model has improved the classification metrics of particularly low-performing classes in our data set as well.

The model parameters employed for training the networks listed above are consolidated in Table 4. It could be inferred that the proposed method is computationally huge when compared to the baseline models. This trade-off in computation vs. accuracy can be considered as the cost of obtaining good inter-class metrics. 

### 4.3. Performance Analysis

The performance of the proposed model is compared against the existing works that include 23 classes for classification, and the results are presented in Table 5. To present a valid comparison between the proposed model and existing works, we have presented the performance analysis with the works that have employed all 23 classes on the HyperKvasir dataset. The proposed work has yielded the best F1-score and accuracy for GI disease classification compared to most of the existing works. This is due to the fusion of significant features from two powerful networks, namely EffficientNet B0 and the proposed Effimix. The integrated features from these networks enabled the proposed model to achieve better inter-class metrics. 

## 5. Conclusions

Gastrointestinal diseases are one of the most prevalent causes of disability in the workforce community. Accurate detection of abnormal tissue growth and other abnormalities plays an important role in determining whether surgical intervention is required. However, the challenges of manually observing each frame received during an endoscopic procedure necessitates the assistance of an AI-powered system. The existing deep learning architectures proposed for gastrointestinal disease classification employ various state-of-the-art CNN models and their combinations. These models mostly apply specific frameworks to improve overall training and loss on the data set. However, there is still room for improvement in terms of overall and class-wise accuracy. In this work, an automated method for gastrointestinal disease classification was proposed. The CNN architecture efficiently aggregates the feature maps from two different models, namely EfficientNet B0 and Effimix. The proposed networks were trained on HyperKvasir benchmark dataset. The proposed model yields an accuracy of 97.99%, with a F1 score, precision, and recall of 97%, 97%, and 98%, respectively on the HyperKvasir dataset. The proposed network can be extended to other gastrointestinal imaging modalities like endoscopic ultrasound (EUS). 

## Figures and Tables

**Table 1:** Distribution of samples for 23 gastrointestinal diseases.

Sl. No.	Class Name	No. of Samples
1	barretts-short	53
2	barretts	41
3	bbps-0-1	646
4	bbps-2-3	1148
5	cecum	1009
6	dyed-lifted-polyps	1002
7	dyed-resection-margins	989
8	esophagitis-a	403
9	esophagitis-b-d	260
10	hemorrhoids	6
11	ileum	9
12	impacted-stool	131
13	polyps	1028
14	pylorus	999
15	retroflex-rectum	391
16	retroflex-stomach	764
17	ulcerative-colitis-grade-0-1	35
18	ulcerative-colitis-grade-1-2	11
19	ulcerative-colitis-grade-1_	201
20	ulcerative-colitis-grade-2-3	28
21	ulcerative-colitis-grade-2	443
22	ulcerative-colitis-grade-3	133
23	z-line	932

**Table 2:** Summary of the ablation studies made.

Sl. No.	Model	Accuracy	Macro Average					Weighted Average				
			F1	Precision	Recall	MCC	Kappa	F1	Precision	Recall	MCC	Kappa
1	EfficientNet B0	0.956	0.95	0.95	0.95	0.954	0.956	0.95	0.95	0.95	0.95	0.95
2	Effimix Network	0.854	0.85	0.85	0.85	0.85	0.856	0.85	0.85	0.85	0.85	0.856
3	Proposed network	0.9799	0.97	0.97	0.98	0.98	0.98	0.97	0.98	0.97	0.98	0.98

**Table 3:** Class-wise metrics of the proposed model.

S. No.	Class Name	EfficientNet B0			Effimix			Combined Model		
		F1	Precision	Recall	F1	Precision	Recall	F1	Precision	Recall
1	barretts-short	0.941	0.932	0.949	0.64	0.71	0.58	0.96	0.97	0.95
2	barretts	0.937	0.91	0.95	0.67	0.77	0.58	0.99	0.94	0.97
3	bbps-0-1	0.97	0.97	0.98	0.98	0.99	0.97	0.99	1	0.99
4	bbps-2-3	0.99	0.99	1	1	1	1	0.99	1	0.99
5	cecum	0.97	1	0.94	0.99	0.99	0.99	1	1	1
6	dyed-lifted-polyps	0.95	0.97	0.94	0.77	0.84	0.81	0.96	1	0.94
7	dyed-resection-margins	0.96	0.95	0.97	0.83	0.78	0.88	0.96	0.94	1
8	esophagitis-a	0.85	0.87	0.83	0.44	0.45	0.43	0.92	0.92	0.91
9	esophagitis-b-d	0.93	0.97	0.89	0.69	0.65	0.74	0.96	0.96	0.97
10	hemorrhoids	1	1	1	0.98	0.97	1	1	1	1
11	ileum	0.99	0.99	1	0.99	0.98	1	1	1	1
12	impacted-stool	0.99	0.99	0.98	0.99	0.99	1	0.99	0.99	1
13	polyps	1	1	1	0.98	0.99	0.98	0.99	1	0.99
14	pylorus	0.96	0.93	1	0.98	0.99	0.97	0.99	0.99	1
15	retroflex-rectum	0.98	0.99	0.97	0.94	0.97	0.91	0.99	0.99	0.98
16	retroflex-stomach	0.98	0.99	0.98	0.92	0.89	0.95	0.99	0.99	0.99
17	ulcerative-colitis-grade-0-1	0.93	0.88	0.99	0.94	0.91	0.96	0.98	0.96	1
18	ulcerative-colitis-grade-1-2	1	1	1	0.99	0.99	0.99	0.99	0.99	1
19	ulcerative-colitis-grade-1_	0.91	0.94	0.88	0.72	0.78	0.67	0.95	0.99	0.91
20	ulcerative-colitis-grade-2-3	0.97	1	0.95	0.94	0.89	0.99	0.99	0.98	1
21	ulcerative-colitis-grade-2	0.87	0.9	0.84	0.66	0.7	0.63	0.95	0.96	0.94
22	ulcerative-colitis-grade-3	0.95	0.92	0.98	0.86	0.8	0.93	0.96	0.92	1
23	z-line	0.88	0.84	0.92	0.6	0.54	0.68	0.95	0.96	0.94
	Average	0.95	0.95	0.95	0.84	0.85	0.85	0.97	0.97	0.97

**Table 4:** Model parameters of the networks trained.

S. No.	Network	Hyper Parameters	Total Number of Trainable Parameters
1	EfficientNet B0	Optimiser: Adam optimiser No. of epochs: 50 Learning rate: 0.001 Weight decay: 0.0001	1,729,176
2	Effimix Network	Optimiser: Adam optimiser No. of epochs: 50 Learning rate: 0.0001 Weight decay: 0.0001	5,288,548
3	Proposed feature fusion network	Optimiser: Adam optimiser No. of epochs: 100 Learning rate: 0.0001 Weight decay: 0.0001	7,041,276

**Table 5:** Performance comparison of the proposed network against existing networks.

S. No.	Source	Method	F1	Precision	Accuracy	Recall
1	He et al. [20]	ResNet-152, MobileNetV3	0.66	0.68	-	0.65
2	Gjestang et al. [30]	Teacher-student framework	0.89	0.89	0.89	-
3	Barbhuiya et al. [22]	DarkNet	0.71	0.71	-	0.74
4	Galdran et al. [2]	CNN—BiT	0.92	-	-	-
5	Galdran et al. [21]	MobileNetV2, ResNeXt-50	0.64	-	0.65	-
6	Proposed work	Novel feature fusion network	0.97	0.97	0.98	0.98

## Data Availability

Not applicable.
